# Dynamic Analysis of an SEIR Model with Distinct Incidence for Exposed and Infectives

**DOI:** 10.1155/2013/871393

**Published:** 2013-05-26

**Authors:** Junhong Li, Ning Cui

**Affiliations:** Department of Mathematics and Sciences, Hebei Institute of Architecture and Civil Engineering, Zhangjiakou, Hebei 075000, China

## Abstract

An SEIR model with vaccination strategy that incorporates distinct incidence rates for the exposed and the infected populations is studied. By means of Lyapunov function and LaSalle's invariant set theorem, we proved the global asymptotical stable results of the disease-free equilibrium. The sufficient conditions for the global stability of the endemic equilibrium are obtained using the compound matrix theory. Furthermore, the method of direct numerical simulation of the system shows that there is a periodic solution, when the system has three equilibrium points.

## 1. Introduction

Mathematical models have become important tools in analyzing the spread and the control of infectious diseases. Many infectious diseases in nature, such as measles, HIV/AIDS, SARS, and tuberculosis (see [1–6]), incubate inside the hosts for a period of time before the hosts become infectious. Li and Fang (see [7]) studied the global stability of an age-structured SEIR model with infectivity in latent period. Yi et al. (see [8]) discussed the dynamical behaviors of an SEIR epidemic system with nonlinear transmission rate. Li and Zhou (see [9]) considered the global stability of an SEIR model with vertical transmission and saturating contact rate.

In this paper, we will consider an SEIR model that the diseases can be infected in the latent period and the infected period. The population size *N* is divided into four homogeneous classes: the susceptible *S*(*t*), the exposed (in the latent period) *E*(*t*), the infective *I*(*t*), and the recovered *R*(*t*). It is assumed that all the offsprings at birth are susceptible to the disease. The inflow rate (including birth and immigration) and outflow rate (including natural death and emigration) are denoted by *b* and *d*, respectively. The rate of disease-caused death is taken as *α*. We assume that susceptible individuals are vaccinated at a constant per capita rate *a*. Due to the partial efficiency of the vaccine, only *σ* fraction of the vaccinated susceptibles goes to the recovered class. The remained 1 − *σ* fraction of the vaccinated susceptibles has no immunity at all and goes to the exposed class after infected by contact with the infectives. If *σ* = 0, it means that the vaccine has no effect at all, and if *σ* = 1, the vaccine is perfectly effective. The positive parameter *ε* is the rate at which the exposed individuals become infectious. *γ* is the constant rate, at which the infectious individuals recover with acquiring permanent immunity. The transfer mechanism from the class *S*(*t*) to the class *E*(*t*) is guided by the function *β*(*I* + *qE*)/*N*, where *β* is the force of infection. *q* denotes the relative measure of infectiousness for the asymptomatic class *E*(*t*).

Based on these considerations, and with reference to [10–12], the SEIR model is given by the following system of differential equations:

(1) $$\begin{array}{c} S^{\prime }=bN-F\left(S,E,I\right)-\left(\sigma a+d\right)S,\\ E^{\prime }=F\left(S,E,I\right)-\left(d+\varepsilon \right)E,\\ I^{\prime }=\varepsilon E-\left(\alpha +\gamma +d\right)I,\\ R^{\prime }=\sigma aS+\gamma I-dR,\\ 0=S+E+I+R-N, \end{array}$$

where the derivative *d*/*dt* is denoted by  ′ and *F*(*S*, *E*, *I*) = *βS*(1 − *a*)(*I* + *qE*)/*N* + *βaS*(1 − *σ*)(*I* + *qE*)/*N*.

Thus, the total population size *N* implies *N*′ = (*b* − *d*)*N* − *αI*. Let *s* = *S*/*N*, *e* = *E*/*N*, *i* = *I*/*N*, and *r* = *R*/*N*.

Because the variable *R* does not appear in the equations of *S*, *E*, and *I*, we only need to consider the following subsystem:

(2) $$\begin{array}{c} s^{\prime }=b-\beta s\left(1-\sigma a\right)\left(i+qe\right)-\left(\sigma a+b\right)s+s\alpha i,\\ e^{\prime }=\beta s\left(1-\sigma a\right)\left(i+qe\right)-\left(b+\varepsilon \right)e+e\alpha i,\\ i^{\prime }=\varepsilon e-\left(\alpha +\gamma +b\right)i+\alpha i^{2},\\ 0=s+e+i+r-1. \end{array}$$

The system (2) is equivalent to (1). From biological considerations, we study (2) in the following closed set:

(3) $$\begin{array}{c} T=\left\{\left(s,e,i\right)\in R_{+}^{3}\;|\;0\leq s+e+i\leq 1\right\}, \end{array}$$

where *R*_+_^3^ denotes the nonnegative cone of *R* ^3^ including its lower dimensional faces.

## 2. Equilibria and Global Stability

It is easy to visualize that (2) always has a disease-free equilibrium *P*_0_(*b*/(*σa* + *b*), 0,0). The Jacobian matrix of (2) at an arbitrary point *P*(*s*, *e*, *i*) takes the following form:

(4) $$\begin{array}{c} J\left(P\right)=\left[\begin{array}{ccc} J_{11} & -q\beta \left(1-\sigma a\right)s & J_{13}\\ J_{21} & J_{22} & J_{23}\\ 0 & \varepsilon & J_{33} \end{array}\right], \end{array}$$

where

(5) $$\begin{array}{c} J_{11}=-\beta \left(1-\sigma a\right)\left(i+qe\right)-\left(\sigma a+b\right)+\alpha i,\\ J_{13}=\alpha s-\beta \left(1-\sigma a\right)s,\\ J_{23}=\alpha e+\beta \left(1-\sigma a\right)s,\\ J_{33}=2\alpha i-\left(\alpha +\gamma +b\right),\\ J_{22}=\beta q\left(1-\sigma a\right)s-\left(b+\varepsilon \right)+\alpha i,\\ J_{21}=\beta \left(1-\sigma a\right)\left(i+qe\right). \end{array}$$

**Theorem 1**

If *R*_0_ < 1, the disease-free equilibrium *P*_0_ is locally asymptotically stable, where

(6) $$\begin{array}{c} R_{0}=\frac{b\beta \left(1-\sigma a\right)\left(q\alpha +q\gamma +qb+\varepsilon \right)}{\left(b+\varepsilon \right)\left(\alpha +b+\gamma \right)\left(\sigma a+b\right)}. \end{array}$$

**Proof**

Let

(7) $$\begin{array}{c} \lambda_{1}=\frac{\left(\varepsilon +q\alpha +q\gamma +qb\right)\left(\alpha +\gamma +2b+\varepsilon \right)}{\left(\alpha +\gamma +b\right)\left(b+\varepsilon \right)qR_{0}}-1. \end{array}$$

We calculate the characteristic equation of *J*(*P*_0_) as follows:

(8) (λ+σa+b) ×(λ2+bqβ(1−σa)λ1λ(σa+b)      +(b+ε)(α+γ+b)(1−R0))=0.

The stability of *P*_0_ is equivalent to all eigenvalues of (8) being with negative real parts, which can be guaranteed by *R*_0_ < 1. Consequently, the disease-free equilibrium is local asymptotical stability. This proves the theorem.

**Theorem 2**

If *R*_01_ ≤ 1, the disease-free equilibrium *P*_0_ is globally asymptotically stable, where

(9) $$\begin{array}{c} R_{01}=\frac{\beta \left(1-\sigma a\right)\left(q\alpha +q\gamma +qb+\varepsilon \right)}{\left(b+\varepsilon \right)\left(\alpha +b+\gamma \right)}. \end{array}$$

**Proof**

Consider the following function:

(10) $$\begin{array}{c} L=\left(\alpha +\gamma +b\right)e+\beta \left(1-\sigma a\right)i. \end{array}$$

Its derivative along the solutions to the system (2) is as follows:

(11) L′=i[(α+γ+b)β(1−σa)s−(1−σa)(α+γ+b−αi)]+e[(α+γ+b)qβ(1−σa)s   −(α+γ+b)(b+ε+αi)+(1−σa)ε]≤e(α+γ+b)(b+ε)(R01−1)≤0.

Furthermore, *L*′ = 0 only if *e* = 0. The maximum invariant set in {(*s*, *e*, *i*) ∈ *T* : *L*′ = 0} is the singleton {*P*_0_}. When *R*_01_ ≤ 1, the global stability of *P*_0_ follows from LaSalle's invariance principle (see [13]). This completes the proof.

**Theorem 3**

Equation (2) has a unique endemic equilibrium P*(s*, e*, i*) if *b* ≥ *α* and Δ > 1, where

(12) Δ=(b+ε−α)(γb+1)×(1ε−(σa+b−α)β(1−σa)(qb+qγ+ε)).

**Proof**

Let the right side of each of the first three differential equations equal to zero in (2); we obtain the following:

(13) $$\begin{array}{c} \beta s^{*}\left(1-\sigma a\right)\left(i^{*}+qe^{*}\right)-\left(b+\varepsilon \right)e^{*}+\alpha e^{*}i^{*}=0,\\ \varepsilon e^{*}-\left(\alpha +\gamma +b\right)i^{*}+\alpha i^{*2}=0,\\ b-\beta s^{*}\left(1-\sigma a\right)\left(i^{*}+qe^{*}\right)-\left(\sigma a+b\right)s^{*}+\alpha s^{*}i^{*}=0, \end{array}$$

with s* > 0, e* > 0 and i* > 0. So we get

(14) $$\begin{array}{c} \frac{b}{s^{*}}=\beta \left(1-\sigma a\right)\left(i^{*}+qe^{*}\right)-\left(\sigma a+b\right)+\alpha i^{*},\\ \frac{\beta s^{*}\left(1-\sigma a\right)\left(i^{*}+qe^{*}\right)}{e^{*}}=\left(b+\varepsilon \right)+\alpha i^{*},\\ \varepsilon e^{*}=\left(\alpha +\gamma +b\right)i^{*}-\alpha i^{*2}. \end{array}$$

When the three equations of (14) are multiplied together, we obtain the following:

(15) bβε(1−σa)[(α+γ+b)qε+1−αqi∗ε] =(b+ε−αi∗)(α+γ+b−αi∗)    ×[−αqβ(1−σa)i∗2ε       +(β(1−σa)(αq+γq+bq+ε)ε+α)i∗       −(σa+b)].

Define the following:

(16) $$\begin{array}{c} f\left(i\right)=f_{1}\left(i\right)\left(b+\varepsilon -\alpha i\right)\left(\alpha +\gamma +b-\alpha i\right),\\ g\left(i\right)=b\beta \varepsilon \left(1-\sigma a\right)\left[\frac{\left(\alpha +\gamma +b\right)q}{\varepsilon }+1-\frac{\alpha qi}{\varepsilon }\right], \end{array}$$

where

(17) f1(i)=[−αqβ(1−σa)i2ε +(β(1−σa)(αq+γq+bq+ε)ε+α)i −(σa+b)],

and the roots of *f*(*i*) are *i*_1_ = (*b* + *ε*)/*α* > 1, *i*_2_ = (*α* + *γ* + *b*)/*α* > 1, and the other two are *i*_3_, *i*_4_ which satisfy *f*_1_(*i*) = 0. *i*_0_ = (*α* + *γ* + *b*)/*α* + *ε*/(*αq*) is the root of *g*(*i*). Direct calculations show the following:

(18) g(1)=bβε(1−σa)(qb+qγ+ε)>0,f(0)=−(σa+b)(b+ε)(α+γ+b)<0,f(1)=Δg(1),    i0>i1,  i0>i2,i3i4=ε(σa+b)αqβ(1−σa)>0,i3+i4=1+b+γα+ε(1αq+1qβ(1−σa))>2.

Since Δ > 1, the linear function *g*(*i*) has exactly one intersection with the function *f*(*i*) where *i* lies in the interval (0,1). Furthermore, s* and e* can be uniquely determined from i* by the following:

(19) $$\begin{array}{c} e^{*}=\frac{i^{*}\left(\alpha +\gamma +b-\alpha i^{*}\right)}{\varepsilon },\\ s^{*}=\frac{b}{\left[\beta \left(1-\sigma a\right)\left(i^{*}+e^{*}\right)+\left(\sigma a+b\right)+\alpha i^{*}\right]}. \end{array}$$

From this, we can easily see that (2) has a unique endemic equilibrium. This completes the proof.

Denote the interior of *T* by $\overset{\circ }{T}$. In this paper, we obtain sufficient conditions that the equilibrium is globally asymptotically stable using the geometrical approach of Li and Muldowney in [14].

**Theorem 4**

The unique endemic equilibrium P* is globally asymptotically stable in $\overset{\circ }{T}$, when

(20) $$\begin{array}{c} b\geq \alpha ,\;\;\Delta >1,\;\;R_{01}>1,\;\;\alpha <\min\left\{\sigma a,b,\varepsilon \right\}. \end{array}$$

**Proof**

Since *R*_01_ > 1, namely, Δ < *R*_0_ and *P*_0_ is unstable, we can easy see that (2) satisfies the assumptions (*H*_1_) and (*H*_2_) (see [14]) in the interior of its feasible region *T*. The unique equilibrium is locally asymptotically stable using simple calculation.

Let *x* = (*s*, *e*, *i*) and *f*(*x*) denotes the vector field of (2) and

(21) $$\begin{array}{c} J^{\left[2\right]}\left(P\right)=\left(\begin{array}{ccc} a_{11} & q\beta s\left(1-\sigma a\right)+\alpha e & -\alpha s+\beta s\left(1-\sigma a\right)\\ \varepsilon & a_{22} & -q\beta s\left(1-\sigma a\right)\\ 0 & \beta \left(1-\sigma a\right)\left(i+qe\right) & a_{33} \end{array}\right), \end{array}$$

where

(22) $$\begin{array}{c} a_{11}=-\beta \left(1-\sigma a\right)\left(i+qe-qs\right)-\left(\sigma a+2b+\varepsilon \right)+2\alpha i,\\ a_{22}=-\beta \left(1-\sigma a\right)\left(i+qe\right)-\left(2b-d+\alpha +\beta \right)+3\alpha i,\\ a_{33}=-q\beta s\left(1-\sigma a\right)-\left(2b+\varepsilon +\alpha +\beta \right)+3\alpha i. \end{array}$$

Set the the following function:

(23) $$\begin{array}{c} P\left(x\right)=P\left(s,e,i\right)=\mathrm{diag}\;\left(1,\frac{e}{i},\frac{e}{i}\right). \end{array}$$

Then the matrix *B* = *P*_*f*_ *P* ^−1^ + *PJ* ^[2]^ *P* ^−1^ can be written in block form as follow:

(24) $$\begin{array}{c} B=\left(\begin{array}{cc} B_{11} & B_{12}\\ B_{21} & B_{22} \end{array}\right), \end{array}$$

where

(25) $$\begin{array}{c} B_{11}=a_{11},\;\;B_{21}={\left(\begin{array}{cc} \frac{\varepsilon e}{i} & 0 \end{array}\right)}^{\prime },\\ B_{12}=\left(\frac{\beta \left(1-\sigma a\right)si}{e}+\alpha i\frac{\left(\beta \left(1-\sigma a\right)-\alpha \right)si}{e}\right),\\ B_{221}=\frac{e^{\prime }}{e}-\frac{i^{\prime }}{i}+a_{22},\;\;B_{222}=\frac{e^{\prime }}{e}-\frac{i^{\prime }}{i}+a_{33},\\ B_{22}=\left(\begin{array}{cc} B_{221} & -q\beta s\left(1-\sigma a\right)\\ \beta \left(1-\sigma a\right)\left(i+qe\right) & B_{222} \end{array}\right). \end{array}$$

Let (*u*, *v*, *w*) denote the vectors in $R^{3}≅R^{\left(\begin{array}{c} 3\\ 2 \end{array}\right)}$, we select a norm in *R* ^3^ as |(*u*, *v*, *w*)| = max⁡⁡{|*u*|, |*v*| + |*w*|} and let *ρ* denote the Lozinskii measure with respect to this norm. Using the method of estimating *ρ* in [15], we have *ρ*(*B*) ≤ sup⁡⁡(*g*_1_, *g*_2_), where

(26) $$\begin{array}{c} g_{1}=\rho_{1}\left(B_{11}\right)+\left|B_{12}\right|,\;\;g_{2}=\left|B_{21}\right|+\rho_{1}\left(B_{22}\right), \end{array}$$

|*B*_12_|, |*B*_21_| are matrix norms with respect to the *l*_1_ vector norm, and *ρ*_1_ denotes the Lozinskii measure with respect to *l*_1_ norm. More specifically, *ρ*_1_(*B*_11_) = *B*_11_, |*B*_21_| = *εe*/*i*, and |*B*_12_| = *β*(1 − *σa*)*si*/*e* + *αi*.

Rewriting the system (2), we have the following:

(27) $$\begin{array}{c} \frac{\varepsilon e}{i}=\frac{i^{\prime }}{i}+\left(\alpha +\gamma +b\right)-\alpha i. \end{array}$$

Therefore,

(28) $$\begin{array}{c} g_{1}=\frac{e^{\prime }}{e}-\beta \left(1-\sigma a\right)\left(i+qe\right)-\left(\sigma a+b\right)+2\alpha i,\\ g_{2}=\frac{e^{\prime }}{e}+2\alpha i-b-\min\left\{\sigma a,\varepsilon \right\}. \end{array}$$

Since *b* ≥ *α* and *α* < min⁡⁡{*σa*, *b*, *ε*}, there is *m* = max⁡⁡{*σa* + *b* − 2*α*, *b* + *ε* − 2*α*} > 0 and

(29) $$\begin{array}{c} \rho \left(B\right)\leq \sup\left(g_{1},g_{2}\right)\leq \frac{e^{\prime }}{e}-m\;\text{for}\;t>\overset{-}{t}. \end{array}$$

Along each solution *x*(*t*, *x*_0_) to (2) such that *x*_0_ ∈ *K*, where *K* is the compact absorbing set, we thus have the following:

(30) $$\begin{array}{c} \frac{1}{t}\int_{0}^{t}\rho \left(B\right)ds\leq \frac{1}{t}\log\frac{e\left(t\right)}{e\left(\overset{-}{t}\right)}+\frac{1}{t}\int_{0}^{\overset{-}{t}}\rho \left(B\right)ds-m\frac{t-\overset{-}{t}}{t}, \end{array}$$

which implies

(31) $$\begin{array}{c} {\overset{-}{q}}_{2}\leq -\frac{m}{2}<0. \end{array}$$

This completes the proof.

## 3. Conclusion

In this paper, we discuss an SEIR model that the diseases can be infected in the latent period and the infected period. The vaccine effectiveness is also taken into account. We investigate the global dynamics of the reduced proportional system. If *R*_01_ ≤ 1, the disease-free equilibrium *P*_0_ is globally asymptotically stable. The unique equilibrium P* of the system (2) is globally asymptotically stable in $\overset{\circ }{T}$, when *b* ≥ *α*, Δ > 1, *R*_01_ > 1, and *α* < min⁡⁡{*σa*, *b*, *ε*}. When *q* = *a* = *γ* = 0, (2) becomes the SEIR model without infectivity in latent and disease-caused death (see [8]). When *q* = 0, (2) becomes the SEIR model without infectious in latent (see [12]). 

The parameters are considered in the following cases:

(32) $$\begin{array}{c} \varepsilon =0.05,\;\;\gamma =0.003,\;\;\alpha =0.002,\;\;\beta =0.05, \end{array}$$

(see [16]), and

(33) $$\begin{array}{c} q=1,\;\;b=0.00001,\;\;\sigma a=0.000001. \end{array}$$

**Figure 1:**
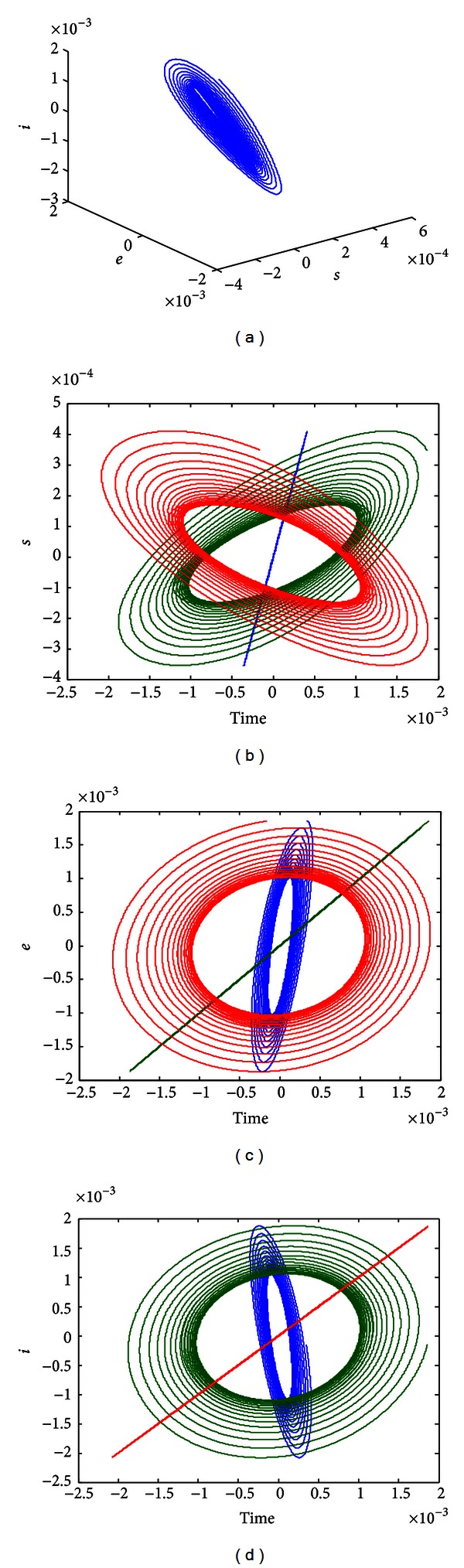
(a) Phase portraits of (2). (b) Time series of *s*. (c) Time series of *e*. (d) Time series of *i*.

At this case, there are three fixed points:

(34) $$\begin{array}{c} E_{1}\left(0.9091,0,0\right),\;\;E_{2}\left(0.0911,0.00018673,0.0019\right),\\ E_{3}\left(0.0000083926,0.025,24.7973\right), \end{array}$$

and *E*_1_, *E*_3_ are unstable, *E*_2_ stable. The method of direct numerical simulation of (2) shows that there is a periodic solution. The phase portraits of the system (2) in Figure 1(a), and time series of *s*, *e*, *i* are given in Figures 1(b), 1(c), and 1(d).
